# Preoperative prognostic nutritional index predicts postoperative infectious complications and oncological outcomes after hepatectomy in intrahepatic cholangiocarcinoma

**DOI:** 10.1186/s12885-021-08424-0

**Published:** 2021-06-16

**Authors:** Tatsuo Matsuda, Yuzo Umeda, Tadakazu Matsuda, Yoshikatsu Endo, Daisuke Sato, Toru Kojima, Kenta Sui, Masaru Inagaki, Tetsuya Ota, Masayoshi Hioki, Masahiro Oishi, Masashi Kimura, Toshihiro Murata, Nobuhiro Ishido, Takahito Yagi, Toshiyoshi Fujiwara

**Affiliations:** 1 Department of Surgery, Tenwakai Matsuda Hospital, Okayama, Japan; 2 Department of Gastroenterological Surgery, Okayama University Graduate School of Medicine, Dentistry, and Pharmaceutical Sciences, 2-5-1 Shikata-cho, Okayama City, 700-8558 Japan; 3 Department of Surgery, Japanese Red Cross Himeji Hospital, Hyogo, Japan; 4 Department of Surgery, Hiroshima City Hiroshima Citizens Hospital, Hiroshima, Japan; 5 Department of Surgery, Okayama Saiseikai General Hospital, Okayama, Japan; 6 Department of Gastroenterological Surgery, Kochi Health Sciences Center, Kochi, Japan; 7 Department of Surgery, National Hospital Organization Fukuyama Medical Center, Hiroshima, Japan; 8 Department of Surgery, National Hospital Organization Okayama Medical Center, Okayama, Japan; 9 Department of Surgery, Fukuyama City Hospital, Hiroshima, Japan; 10 Department of Surgery, Tottori Municipal Hospital, Tottori, Japan; 11 Department of Surgery, Matsuyama Shimin Hospital, Ehime, Japan; 12 Department of Surgery, Onomichi Municipal Hospital, Hiroshima, Japan; 13 Department of Surgery, Japanese Red Cross Kobe Hospital, Hyogo, Japan

**Keywords:** Intrahepatic cholangiocarcinoma, Postoperative complication, Prognostic nutritional index

## Abstract

**Background:**

In the surgical treatment of intrahepatic cholangiocarcinoma (ICC), postoperative complications may be predictive of long-term survival. This study aimed to identify an immune-nutritional index (INI) that can be used for preoperative prediction of complications.

**Patients and methods:**

Multi-institutional data from 316 patients with ICC who had undergone surgical resection were retrospectively analysed, with a focus on various preoperative INIs.

**Results:**

Severe complications (Clavien-Dindo grade III–V) were identified in 66 patients (20.8%), including Grade V complications in 7 patients (2.2%). Comparison of areas under the receiver operating characteristic curve (AUCs) among various INIs identified the prognostic nutritional index (PNI) as offering the highest predictive value for severe complications (AUC = 0.609, cut-off = 50, *P* = 0.008). Multivariate analysis revealed PNI <  50 (odds ratio [OR] = 2.22, *P* = 0.013), hilar lesion (OR = 2.46, *P* = 0.026), and long operation time (OR = 1.003, *P* = 0.029) as independent risk factors for severe complications. In comparing a high-PNI group (PNI ≥ 50, *n* = 142) and a low-PNI group (PNI <  50, *n* = 174), the low-PNI group showed higher rates of both major complications (27% vs. 13.4%; *P* = 0.003) and infectious complications (14.9% vs. 3.5%; *P* = 0.0021). Furthermore, median survival time and 1- and 5-year overall survival rates were 34.2 months and 77.4 and 33.8% in the low-PNI group, respectively, and 52.4 months and 89.3 and 47.5% in the high-PNI group, respectively (*P* = 0.0017).

**Conclusion:**

Preoperative PNI appears useful as an INI correlating with postoperative severe complications and as a prognostic indicator for ICC.

**Supplementary Information:**

The online version contains supplementary material available at 10.1186/s12885-021-08424-0.

## Core tip

This is the first large-scale, multicentre retrospective study to investigate immune-nutritional indices predicting postoperative complications among patients with surgically resected intrahepatic cholangiocarcinoma (ICC). We retrospectively examined the medical records of 316 patients and evaluated preoperative prognostic nutritional index (PNI), neutrophil-to-lymphocyte ratio, lymphocyte-to-monocyte ratio, and controlling nutritional status. Low PNI (PNI < 50) was an independent predictor of severe complications and poor survival. Clinicians should thus pay attention to perioperative care for patients with ICC and low PNI.

## Introduction

Intrahepatic cholangiocarcinoma (ICC) is the second most common primary cancer of the liver, and arises from the intrahepatic bile ducts [1, 2]. Both the incidence and mortality of ICC have been increasing worldwide [1]. ICC has an estimated 5-year survival rate of 30% [3, 4], and represents an aggressive cancer type that can be curatively treated by complete surgical resection. The surgical approach typically involves liver resection with en bloc resection of regional lymph nodes. This represents one of the most invasive surgical procedures for the treatment of a gastrointestinal cancer. Gaya et al. reported on postoperative complications as independent predictors of poor long-term survival in patients with ICC [5]. They reported that 15.6 and 26.2% of patients developed major and minor postoperative complications, respectively. Moreover, 3.5% of patients died within 90 days after surgery.

The tumour-node-metastasis staging system is the standard tool for predicting prognosis in patients with cancer. Both immune and nutritional statuses reportedly play important roles in cancer progression and prognosis [6, 7]. Furthermore, researchers have investigated the associations of various parameters, including prognostic nutritional index (PNI), neutrophil-to-lymphocyte ratio (NLR), lymphocyte-to-monocyte ratio (LMR), and Controlling Nutritional Status (CONUT) score, with postoperative complications and prognosis in patients with cancer. More detailed investigations are thus needed to clarify the relationship between preoperative immune-nutritional indices (INIs) and postoperative complications, to allow the identification of high-risk patients with ICC prior to radical surgery. We therefore aimed to identify INIs predictive of postoperative complications following ICC resection. This appears to represent the first large-scale, multicentre retrospective study to evaluate INIs predicting postoperative complications in patients with surgically resected ICC.

## Materials and methods

### Patients and methods

We retrospectively examined the medical records of 415 patients with ICC who underwent curative surgical resection at 17 institutions in Japan between January 2000 and December 2016. These institutions comprised Okayama University Hospital, Okayama Saiseikai General Hospital, Hiroshima Citizens Hospital, Kochi Health Sciences Center, Himeji Red Cross Hospital, National Fukuyama Medical Center, Tottori Municipal Hospital, Tenwakai Matsuda Hospital, National Okayama Medical Center, Fukuyama City Hospital, Himeji St. Maria Hospital, Matsuyama Municipal Hospital, Sumitomo Besshi Hospital, Onomichi Municipal Hospital, National Iwakuni Medical Center, Himeji Central Hospital, and Kobe Red Cross Hospital. Twelve of these institutions are board-certified training institutions for the Hepatobiliary and Pancreatic Surgery program in Japan [8]. Consequently, most patients were recruited from qualified programs, leading to relatively standardised operative procedures and classification of outcomes. Subjects meeting the following criteria were excluded: 1) non-curative (residual tumor, peritoneal dissemination, or positive surgical margin) surgery; or 2) lacking sufficient data about aforementioned INIs were lacking; or 3) laparoscopic procedure. After excluding those individuals, a total of 316 patients were included in this study. Median patient’s follow-up period after surgery was 27.5 months (interquartile range. 13.2–51.2 months). We examined clinicopathological characteristics, surgical procedures, postoperative complications, overall survival (OS), and PNI, NLR, LMR, and CONUT score as preoperative INIs.

PNI was calculated using the following formula:

$$ 10\times \mathrm{serum}\ \mathrm{albumin}\ \left(\mathrm{g}/\mathrm{dL}\right)+0.005\times \mathrm{total}\ \mathrm{lymphocyte}\ \mathrm{count}\ \left(/{\mathrm{mm}}^3\right) $$

NLR was determined by dividing the neutrophil count by the lymphocyte count. In contrast, LMR was determined by dividing the lymphocyte count by the monocyte count. CONUT score was calculated from serum albumin concentration, total cholesterol concentration, and total peripheral lymphocyte count as previously described [9]. Postoperative complications were defined as any in-hospital or 90-day postoperative complications, graded based on the Clavien-Dindo classification [10]. If a case displayed more than one complication, the complication with the highest grade was used. Infectious complications were categorised according to the Centers for Disease Control Classification System [11]. This study was approved by the Okayama University Hospital Institutional Ethics Board. The need for written informed consent was waived because of the retrospective design.

### Statistical analysis

Clinical variables were compared using the Mann-Whitney U test for continuous data and Pearson’s correlation coefficient for categorical data. Continuous variables are presented as median and interquartile range. Values of *P*< 0.05 were considered significant. The predictive value of potential factors for severe complications (defined as grade III–V complications) was assessed by the corresponding area under the receiver operating characteristic curve (AUC). Youden index was utilised to choose optimal cut-off values, set as the value maximising the sum of sensitivity and specificity. AUCs were compared with each other in a nonparametric approach using the theory for generalised U-statistics to generate an estimated covariance matrix [12]. With regard to survival analysis, survival curves were estimated using Kaplan-Meier methods, and differences in survival were evaluated with the log-rank test and Wilcoxon test. We used logistic regression analysis to identify risk factors for severe complications. For this analysis, clinical variables showing values of *P*< 0.05 in univariate analyses were entered into the multivariate analysis. Odds ratios (ORs) and 95% confidence intervals (CIs) were calculated. All statistical analyses were performed using JMP version 14 (SAS Institute, Cary, NC, USA).

## Results

### Postoperative complications (Clavien-Dindo classification)

All postoperative complications are summarised in Table 1. Postoperative complications occurred in 111 patients (35.2%), of whom 59 patients (18.6%) experienced postoperative complications of grade III–IV. The leading cause of complications was bile leakage (*n*=37, 11.7%), followed by intra-abdominal abscess (*n*=16, 5.1%), and delayed gastric emptying (*n*=15, 4.7%) due to dissection of the lymphatic station around the lesser curvature of the stomach in cases of left-side predominant ICC [13]. In addition, 7 patients (2.2%) died due to Grade V complications, comprising portal vein thrombosis (*n*=4), intra-abdominal abscess (*n*=1), gastrointestinal haemorrhage (*n*=1), and intra-abdominal haemorrhage after rupture of pseudo-aneurysm (*n=*1). The most common infectious complication was surgical site infection (*n*=24, including 16 cases of intra-abdominal abscess), followed by bloodstream infection (*n*=3), intra-abdominal haemorrhage due to abscess(*n=*3), and pneumonia (*n=*1). Of these, severe complications (*Clavien-Dindo* classification grades III-V) were seen in 19 patients, with intra-abdominal abscess being the leading cause.

**Table 1:** Summary of all postoperative complications

Complications	Total number (%)	Grade of surgical complication^**a**^			
		None	I-II	III-IV	V
No complications - Grade II (%)	250 (79.1%)	205 (64.8%)	45 (14.2%)		
Grade III-V (%)	66 (20.8%)			59 (18.6%)	7 (2.2%)
Total no. of complications (%)	111 (35.2%)				
**Cardiovascular**					
heart failure	2 (0.6%)		1 (0.3%)	1 (0.3%)	
deep venous thrombosis	2 (0.6%)		2 (0.6%)		
**Pulmonary**					
pleural effusion	4 (1.3%)		2 (0.6%)	2 (0.6%)	
pneumonia	1 (0.3%)		1 (0.3%)		
**Gastrointestinal**					
gastrointestinal hemorrhage	4 (1.3%)		2 (0.6%)	1 (0.3%)	1 (0.3%)
delayed gastric empting	15 (4.7%)		14 (4.4%)	1 (0.3%)	
intestinal obstruction	5 (1.6%)		2 (0.6%)	3 (0.9%)	
**Surgical site infections**					
superficial-deep wound	8 (2.5%)		7 (2.2%)	1 (0.3%)	
intra-abdominal abscess	16 (5.1%)		1 (0.3%)	14 (4.4%)	1 (0.3%)
**Liver/Biliary**					
bile leak	37 (11.7%)		6 (1.9%)	31 (9.8%)	
portal vein thrombosis	4 (1.3%)				4 (1.3%)
anastomotic leak	1 (0.3%)			1 (0.3%)	
**Others**					
intra-abdominal hemorrhage	3 (0.9%)		1 (0.3%)	1 (0.3%)	1 (0.3%)
sepsis	3 (0.9%)		2 (0.6%)	1 (0.3%)	
ascites	6 (1.9%)		4 (1.3%)	2 (0.6%)	

^a^Clavien-Dindo classification

### Predictive values of INIs for postoperative complications

**Fig. 1:**
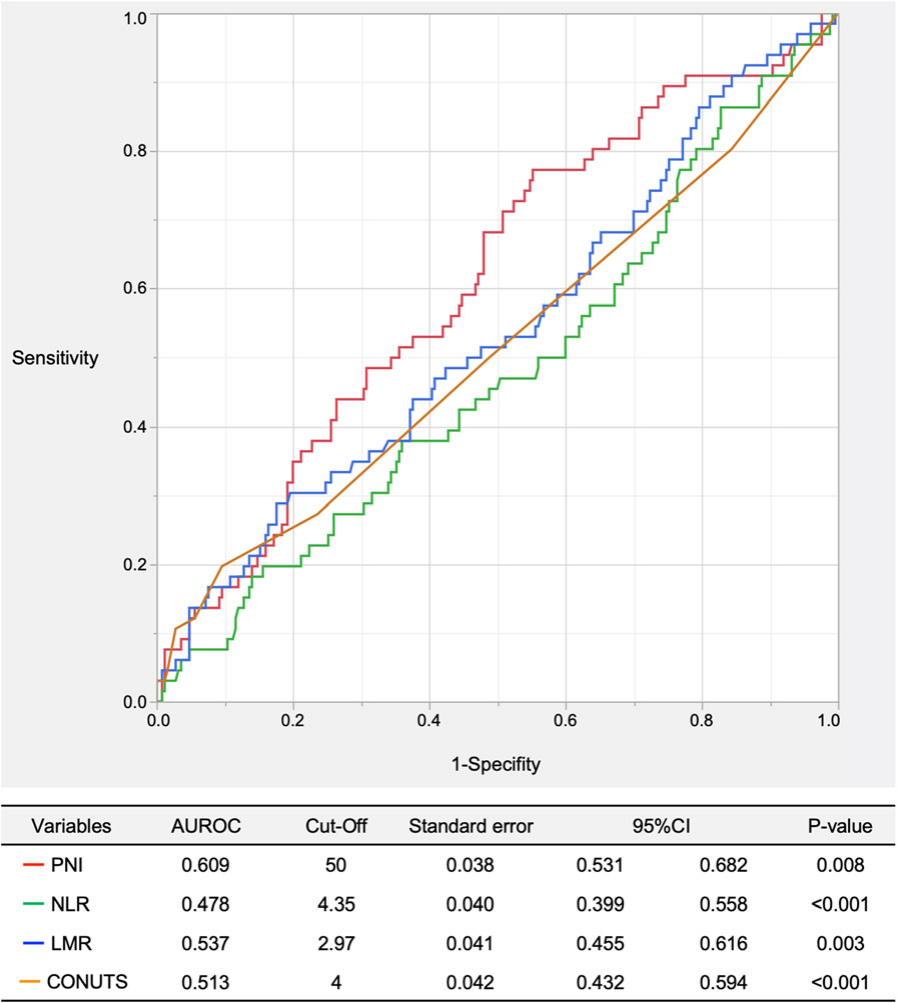
Receiver operating characteristic curves for immune-nutritional indices related to severe postoperative complications

We plotted the receiver operating characteristic curves for PNI, NLR, LMR, and CONUT score to investigate the ability of INIs to predict severe complications. PNI showed the highest predictive value (AUC=0.6094, 95%CI=0.5315–0.6822, *P=*0.008), followed by LMR (AUC=0.537, 95%CI=0.455–0.616, *P=*0.003), CONUT score (AUC=0.518, 95%CI=0.432–0.594, *P*< 0.001), and NLR (AUC=0.478, 95%CI=0.399–0.558, *P*< 0.001). With PNI, the cut-off was calculated as 50, corresponding to the maximal Youden index (Fig. 1). Comparisons of each AUC using a nonparametric approach revealed PNI as the most suitable parameter for predicting postoperative complications (Table 2).

**Table 2:** Comparisons of each AUC for predicting postoperative complications

Variables	difference	Standard error	95%CI		×2	*P*-value
PNI vs NLR	0.131	0.043	0.045	0.215	9.061	0.003
PNI vs LMR	0.073	0.039	−0.004	0.149	3.415	0.064
PNI vs CONUTS	0.095	0.028	0.040	0.151	11.394	< 0.001
LMR vs NLR	0.058	0.042	−0.025	0.141	1.888	0.169
LMR vs CONUTS	0.023	0.044	−0.064	0.109	0.278	0.600
NLR vs CONUTS	−0.035	0.045	−0.123	0.052	0.617	0.431

### Relationships between PNI and clinicopathological characteristics

**Fig. 2:**
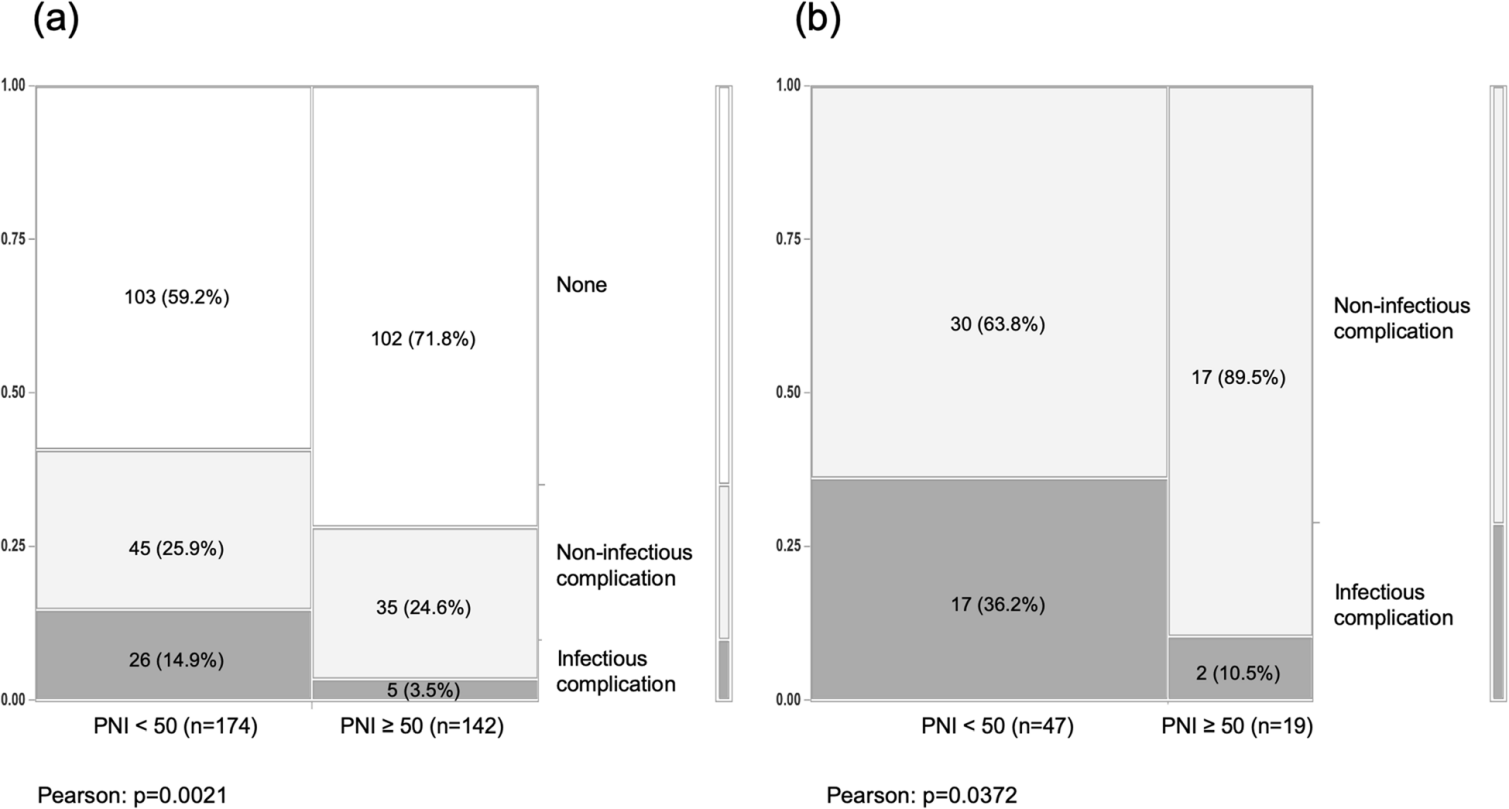
**a**. Frequency of postoperative infectious complications in all patients (*n=*316). **b**. Frequency of postoperative infectious complications in patients with severe complications (*n=*66)

Patients were divided into two groups: a high-PNI group (PNI ≥ 50, *n*=142); and a low-PNI group (PNI < 50, *n*=174). Table 3 outlines the clinicopathological characteristics of the two groups. The low-PNI group showed significantly older age, lower BMI, lower lymphocyte count, lower albumin concentration, longer prothrombin time, lower total cholesterol concentration, higher C-reactive protein concentration, and higher concentration of cancer antigen 19–9. In terms of cancer localisation, hilar-type ICC predominated in the low-PNI group. As a result, bile duct resection was required at the time of surgery more frequently than in the high-PNI group. With regard to postoperative complications, frequency of severe (grade III–V) complications was higher in the low-PNI group than in the high-PNI group (*P=*0.003). Postoperative mortality (grade V) was seen in 7 cases (4%) in the low-PNI group and in 1 case (0.7%) in the high-PNI group. Infectious complications characterised by surgical site infection (including superficial and organ/space abscess), bloodstream infection, and pneumonia were more frequently in the low-PNI group (14.9%) than in the high-PNI group (3.5%; *P*=0.021). On the other hand, frequencies of non-infectious complications characterised by other events were comparable between the low-PNI group (25.9%) and the high-PNI group (24.6%) (Table 3, Fig. 2a). For severe complications (*n*=66), the frequency of infectious complications was 36.2% in the low-PNI group, but only 10.5% in the high-PNI group (*P=*0.0372) (Fig. 2b). Adjuvant chemotherapy resulted in a significantly lower induction rate in the low-PNI group (32.7%) than in the high-PNI group (45.7%; *P=*0.0181) (Table 3).

**Table 3:** Clinicopathological characteristics of the low PNI group and the high PNI group

Variables	All patients (***n***=316)	Low PNI group	High PNI group	P-value*
		PNI < 50 (***n=***174)	PNI ≥ 50 (***n=***142)	
**Preoperative factors**				
Male, n (%)	187 (59.1%)	100 (57.5%)	87 (61.3%)	0.495
Age (years), median (IQR)	71 (63–76)	72.5 (66–79)	68 (62–74)	0.002
BMI, median (IQR)	22.1 (20.0–24.8)	22.0 (19.2–24.0)	22.8 (21.0–25.2)	0.048
Lymphocytes (/ul), median (IQR)	1560 (1190-1931)	1328 (1058-1625)	1894 (1508-2332)	<.0001
Neutrophils (/ul), median (IQR)	3652 (2834-4908)	3692 (2781-5027)	3634 (2920-4474)	0.759
Monocytes (/ul), median (IQR)	342 (277–440)	342 (270–436)	342 (281–442)	0.669
Platelet count (104/uL), median (IQR)	19.9 (15.4–24.5)	19.9 (14.5–25.4)	20.1 (16.8–23.6)	0.827
Total Bilirubin (mg/dl), median (IQR)	0.7 (0.5–0.9)	0.7 (0.50–0.90)	0.7 (0.54–0.90)	0.698
Albumin (mg/dl), median (IQR)	4.1 (3.8–4.4)	3.8 (3.5–4.1)	4.3 (4.3–4.6)	<0.001
AST (U/L), median (IQR)	29 (22–39)	29 (22–42)	29 (23–37)	0.978
ALT (U/L), median (IQR)	25 (16–39)	24 (15–39)	27 (18–38)	0.142
Prothrombin time (INR), median (IQR)	1.03 (0.97–1.10)	1.04 (1.0–1.13)	1.01 (0.95–1.07)	<.0001
Total Cholesterol (mg/dL), median (IQR)	185 (166–211)	180 (156–206)	194 (173–220)	0.015
HBV-Ag, n (%)	18 (5.7%)	11 (6.3%)	7 (4.9%)	0.595
HCV-Ab, n (%)	47 (14.9%)	29 (16.7%)	18 (12.7%)	0.321
CRP (mg/dl), median (IQR)	0.2 (0.09–0.70)	0.3 (0.10–1.04)	0.16 (0.08–0.34)	0.010
Preoperative chemotherapy, n (%)	7 (2.2%)	6 (3.5%)	1 (0.7%)	0.099
**Tumor factor**				
Morphology, n (%)				
Mass-forming (MF)	234 (74.5%)	123 (71.1%)	111 (78.7%)	0.260
Periductal-infiltrating (PI)	30 (9.6%)	16 (9.3%)	14 (9.9%)	
MF+PI	34 (10.8%)	23 (13.3%)	11 (7.8%)	
Intraductal Growth	16 (5.1%)	11 (6.3%)	5 (3.6%)	
Tumor size (cm), median (IQR)	4.0 (2.8–6.5)	4.5 (3.0–7.0)	4.0 (2.5–6.0)	0.253
Multi-nodular, n (%)	63 (19.9%)	35 (20.1%)	28 (19.7%)	0.930
Localization, n (%)				
Hilar	111 (35.1%)	72 (41.4%)	39 (27.5%)	0.010
Peripheral	205 (64.9%)	102 (58.6%)	103 (72.5%)	
CEA (ng/ml), median (IQR)	2.90 (1.80–5.87)	3.1 (2.0–7.2)	2.8 (1.6–4.7)	0.323
CA19–9 (U/ml), median (IQR)	39.4 (14.3–246.9)	52.1 (15.8–356.2)	26.3 (14.1–127.9)	0.024
**Operative factors**				
Major hepatectomy, n (%)	222 (70.3%)	128 (73.5%)	94 (66.2%)	0.154
Type of hepatectomy, n (%)				
Segmentectomy/Sub-segmentectomy	82 (25.9%)	39 (22.4%)	43 (30.3%)	0.196
Hemihepatectomy	221 (69.9%)	126 (72.4%)	95 (66.9%)	
Trisectionectomy	13 (4.1%)	9 (5.2%)	4 (2.82%)	
Lymphnode dissection, n (%)	218 (68.9%)	117 (67.2%)	101 (71.1%)	0.458
Bile duct resection, n (%)	82 (25.9%)	53 (30.4%)	29 (20.4%)	0.043
Vascular reconstruction**, n (%)	24 (7.5%)	16 (9.2%)	8 (5.6%)	0.235
Blood loss (ml), median (IQR)	670 (350–1208)	780 (380–1190)	640 (304–1250)	0.499
Operation time (min), median (IQR)	348 (270–448)	354 (271–465)	333 (264–420)	0.228
**Pathological factors**				
Serosa invasion, n (%)	112 (35.4%)	58 (33.3%)	54 (38.0%)	0.386
Vascular invasion, n(%)	160 (50.6%)	87 (50.0%)	73 (51.4%)	0.947
Lymph node metastasis, n(%)	86 (27.2%)	47 (27.0%)	39 (27.5%)	0.528
Differentiation, n (%)				0.221
Well	66 (20.9%)	37 (21.3%)	29 (20.4%)	
Moderate	167 (52.9%)	84 (48.3%)	83 (58.4%)	
Poorly	58 (18.4%)	36 (20.7%)	22 (15.5%)	
Un-classified	25 (7.9%)	17 (8.7%)	8 (5.6%)	
Background liver, n (%)				0.302
Normal	234 (74.0%)	123 (70.7%)	111 (78.2%)	
Hepatitis	60 (19.0%)	38 (21.8%)	22 (15.5%)	
Fibrosis	22 (7.0%)	13 (7.5%)	9 (6.3%)	
**Post-operative factors**				
Clavien-Dindo classification, n (%)				0.019
none	205 (64.9%)	103 (59.2%)	102 (71.8%)	
Grade I-II	45 (14.2%)	24 (13.8%)	21 (14.8%)	
Grade III-IV	58 (18.4%)	40 (23.0%)	18 (12.7%)	
Grade V	8 (2.5%)	7 (4.0%)	1 (0.7%)	
All Complication. CD-Grade I-V	111 (35.1%)	71 (40.8%)	40 (28.2%)	0.019
All Complication. CD-Grade III-V	66 (20.9%)	47 (27.0%)	19 (13.4%)	0.003
Infectious Complication. CD-Grade I-V	31 (9.8%)	26 (14.9%)	5 (3.5%)	0.002
Infectious Complication. CD-Grade IIII-V	19 (6.0%)	17 (36.2%) ***	2 (10.5%) ***	0.037
Adjuvant chemotherapy, n (%)	122 (38.6%)	57 (32.7%)	65 (45.7%)	0.018

* Low-PNI group vs High-PNI group

** reconstruction of portal vein or hepatic artery or hepatic vein or inferior vene cava

*** proportion in complicated cases

### Multivariate analysis of risk factors for severe complications

We conducted logistic regression analysis to examine risk factors for major postoperative complications (Table 4). In uni- and multivariate analyses, PNI < 50 (OR=2.22, 95%CI=1.18–4.20, *P=*0.013), hilar-type ICC (OR=2.46, 95%CI=1.11–5.45, *P=*0.026), and longer operation time (OR=1.003, 95%CI=1.000–1.005, *P=*0.029) were revealed as independent risk factors for severe complications. No other preoperative parameters, pathological factors including vascular invasion, lymph node metastasis and differentiation, or operative factors had any predictive impact on the occurrence of severe postoperative complications. In subgroup analysis stratified by tumor location and bile duct resection, major postoperative morbidity in hilar-type ICC was higher in patients with PNI < 50 than in those with PNI ≥ 50 (Supplementary Fig. 1).

**Table 4:** Logistic regression analysis to examine risk factors for major postoperative complications

Variables	Univariate analysis				Multivariate analysis			
	Odds ratio	95% C.I.		***P***-value	Odds ratio	95% C.I.		***P***-value
**Preoperative factors**								
Age: ≥80 vs < 80 (years)	1.965	1.009	3.828	0.046	1.575	0.752	3.298	0.228
BMI	0.959	0.886	1.037	0.291				
Platelet count (10^4^/uL)	1.016	0.981	1.051	0.370				
Total Bilirubin (mg/dl)	1.062	0.863	1.309	0.566				
PNI: < 50 vs ≥ 50	2.395	1.331	4.312	0.002	2.072	1.143	3.961	0.027
AST (U/L)	1.001	0.998	1.005	0.308				
ALT (U/L)	1.003	0.998	1.009	0.183				
Prothrombin time (INR)	1.106	0.184	6.623	0.912				
Total Cholesterol (mg/dL)	1.001	0.995	1.006	0.717				
CRP (mg/dl)	1.082	0.970	1.207	0.162				
Preoperative chemotherapy	2.928	0.639	13.42	0.167				
**Tumor factor**								
Morphology: Mass-forming vs the other type	0.806	0.440	1.479	0.487				
Tumor size (cm)	1.049	0.957	1.151	0.298				
Multi-nodular	1.382	0.724	2.637	0.326				
Localization: Hilar vs Peripheral	2.523	1.151	5.530	0.020	2.548	1.143	5.683	0.022
CEA (ng/ml)	1.003	0.992	1.013	0.608				
CA19–9 (U/ml)	1.000	0.999	1.000	0.827				
Vascular invasion*	1.172	0.680	2.020	0.566				
Lymph node metastasis	1.122	0.579	2.171	0.732				
Tumor differentiation: mod/por vs well	1.662	0.889	3.111	0.112				
**Operative factors**								
Major hepatectomy vs Minor hepatectomy	1.570	0.832	2.962	0.153				
Lymph node dissection	1.254	0.686	2.294	0.460				
Bile duct resection	3.456	1.948	6.129	0.001	1.135	0.491	2.625	0.767
Vascular reconstruction**	2.473	1.030	5.937	0.042	1.061	0.393	2.870	0.906
Blood loss (ml)	1.001	1.000	1.001	0.032	1.000	0.999	1.000	0.612
Operation time (min)	1.004	1.002	1.006	0.001	1.003	1.000	1.006	0.026

*vascular invasion: pathologically diagnosed as invasion to portal vein, hepatic artery, hepatic vein, and inferior vena cava

**reconstruction including portal vein, hepatic artery, hepatic vein, and inferior vena cava

### Prognostic impact of PNI and postoperative complications on survival after surgery

**Fig. 3:**
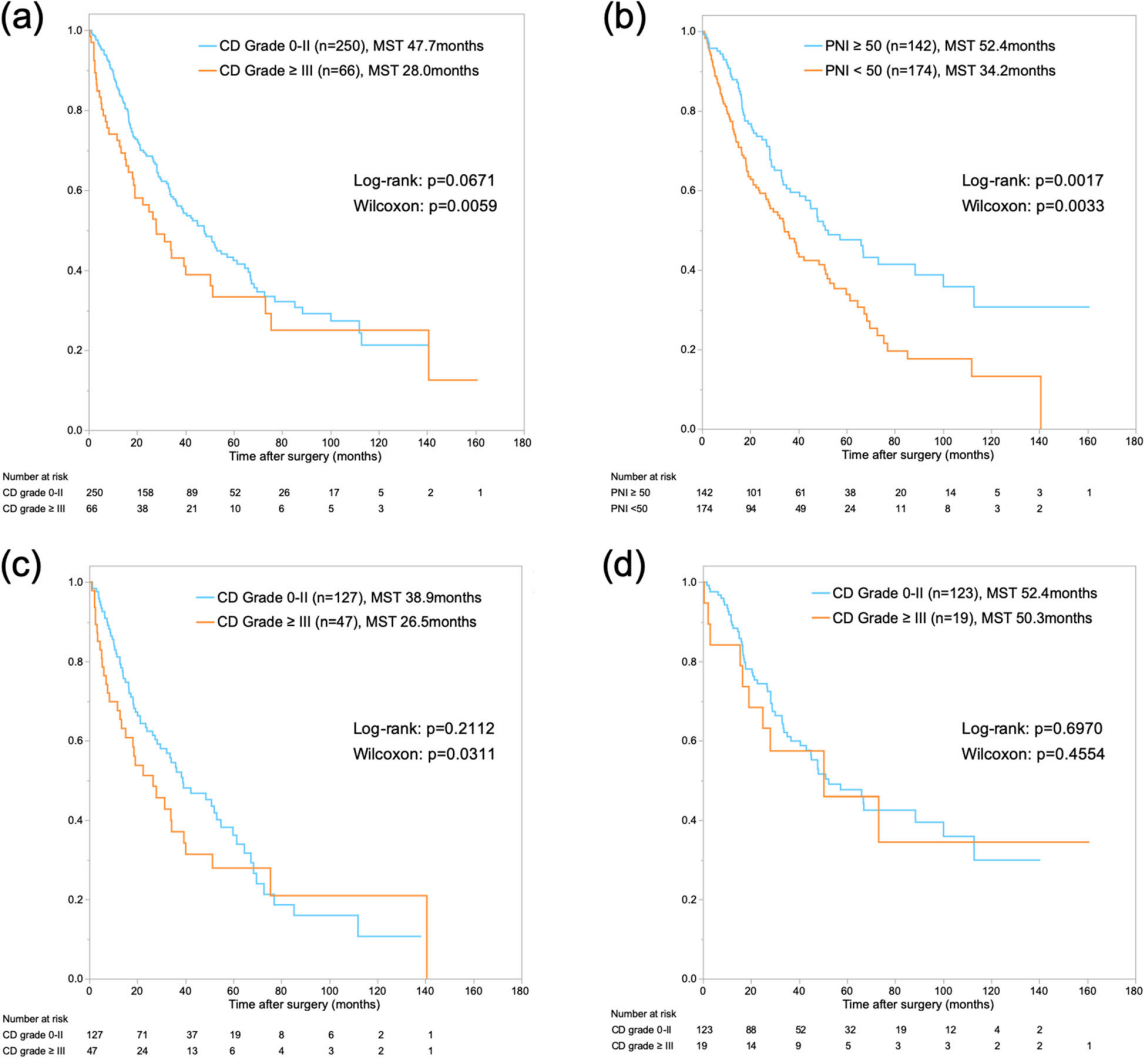
**a**. Kaplan-Meier curves for postoperative overall survival, stratified by grade of postoperative complications. **b**. Kaplan-Meier curves for postoperative overall survival, stratified by PNI. **c**. Kaplan-Meier curves for postoperative overall survival, stratified by grade of postoperative complications in the low-PNI group. **d**. Kaplan-Meier curves for postoperative overall survival, stratified by grade of postoperative complications in the high-PNI group

**Fig. 4:**
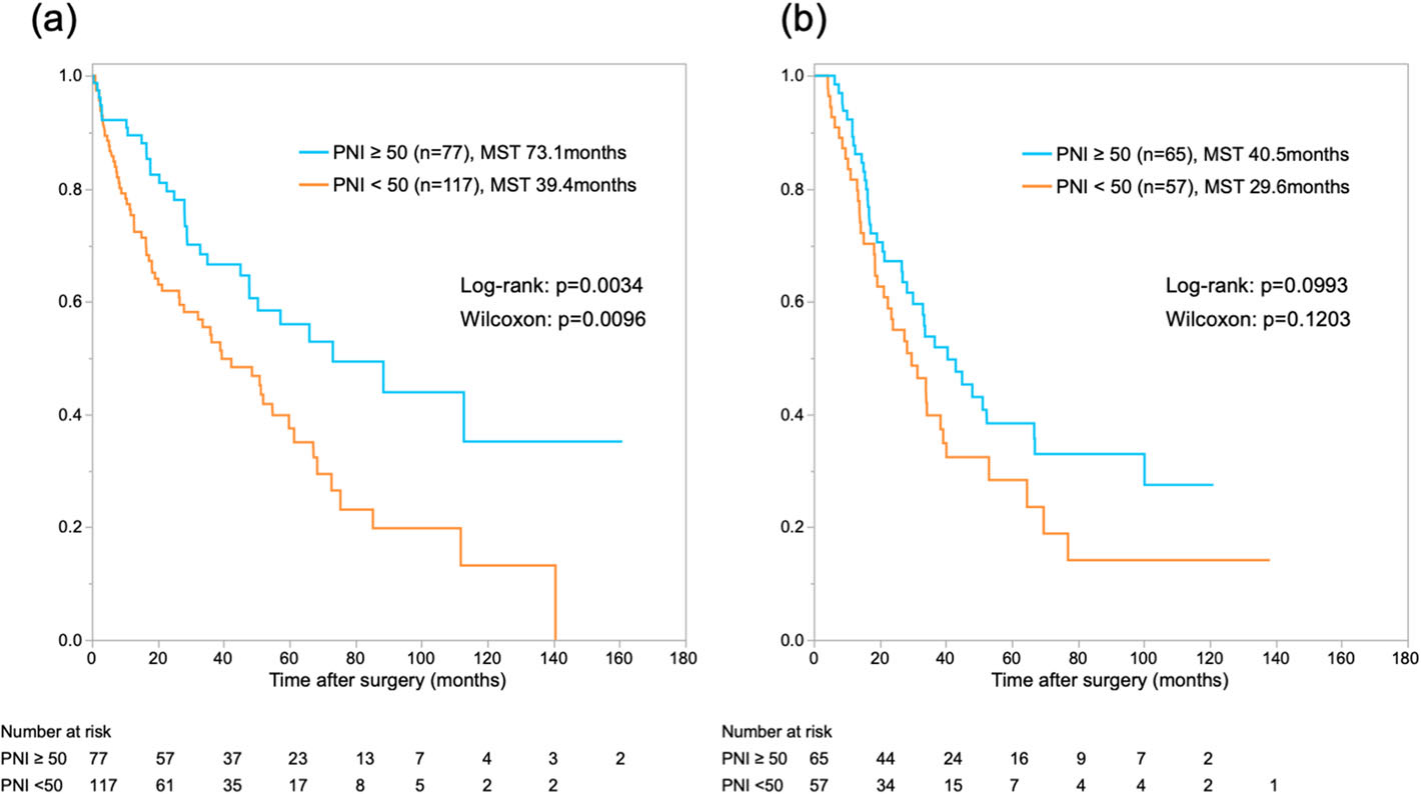
**a**. Kaplan-Meier curves for postoperative overall survival in patients without adjuvant chemotherapy (*n=*194). **b**. Kaplan-Meier curves for postoperative overall survival in patients with adjuvant chemotherapy (*n*=122)

In survival analyses, median survival time (MST) and 1- and 5-year OS rates after surgery were 42.3 months and 82.9 and 40.4%, respectively, for the total patient cohort. In addition, 1- and 5-year recurrence-free survival rates were 59.5 and 29.3%, respectively (Supplementary Fig. 2a, b). Patients with severe (grade III–V) complications showed worse outcomes than patients with grade 0–II complications (MST: 28.0 months vs. 47.7 months; *P=*0.0059) (Fig. 3a). MST was 34.2 months in the low-PNI group, and 52.4 months in the high-PNI group (*P=*0.0017) (Fig. 3b). In subgroup analysis for the low-PNI group, severe (grade III–V) complications were associated with worse short-term outcomes than grade 0–II complications, as in the overall analysis (Fig. 3c). On the other hand, in subgroup analysis for the high-PNI group, grade of complication did not have any impact on short- or long-term outcomes. In other words, patients with high PNI could be expected to overcome severe complications and show a prognosis comparable to that of an uncomplicated case (Fig. 3d). Recurrence-free survival showed a similar trend to the survival analysis for PNI and severe complications (Supplementary Fig. 2c, d). Furthermore, in the subgroup analysis of the patients with and without adjuvant chemotherapy, the low-PNI group showed worse outcomes than the high-PNI group, independent from adjuvant setting (Fig. 4a,b).

## Discussion

Surgical resection is the only curative treatment option for patients with ICC, but rates of morbidity and mortality remain high. Even with curative resection, patients who experience postoperative complications display a poor prognosis [5]. Identification of parameters to effectively predict postoperative complications prior to surgery is thus a critical issue. The present examination of preoperative INIs identified PNI as a useful predictive marker to distinguish high-risk patients with ICC before surgery.

PNI is a widely used systemic inflammation-based prognostic score, simply calculated based on the total lymphocyte count and the concentration of serum albumin in peripheral blood. Both parameters are normally analysed during preoperative examinations [14, 15]. While albumin is the most studied value associated with nutritional status, the lymphocyte count reflects the immune status of a patient. PNI thus represents both the immune and nutritional statuses of the patient. Malnutrition is closely associated with a high risk of postoperative complications [16]. Low PNI has been considered a predictor of postoperative complications for surgeries involving the gastrointestinal tract [17, 18]. A low PNI has recently been reported as a negative prognostic factor for various types of cancers [14, 15, 17, 19]. However, this value has not been well investigated for patients with ICC. Özgür et al. first reported PNI < 40 as an independent and negative prognostic factor for patients with ICC who undergo curative surgical resection [20]. A low preoperative PNI was associated with a poor or undifferentiated ICC phenotype and advanced tumour stage (T2–T4) [20]. Patients with PNI < 50 experienced significantly worse prognosis than those with a higher PNI.

Several reports have been published on INIs in patients with ICC. Miyata et al. reported on the prognostic impact of CONUT score in patients with ICC [21]. A high CONUT score was identified as an independent predictor of poor prognosis, but was not associated with postoperative complications. Gomes et al. reported a high preoperative NLR as a predictor of poorer disease-free survival, associated with tumour aggressiveness characterised by large tumour size, satellite lesions, microvascular invasion, and lymph node involvement [22]. Lin et al. reported on the association between an elevated NLR and poor anti-tumour immunity and low density of tumour-infiltrating CD3+ T cells. NLR could thus represent a marker of poor prognosis for patients with ICC [23].

We classified low- and high-PNI groups on the basis of a cut-off of 50, set for the receiver operating characteristic curve analysis. Other reports have suggested a PNI cut-off between 40 and 50, consistent with the value used in the present study [24]. A low PNI predicts not only postoperative complications, but also poor survival. Nonetheless, severe complications themselves did not represent a significant predictor of poor long-term survival (Fig. 3a). The association between PNI and nutritional and immune statuses might reflect tumour progression and aggressiveness through the lymphocyte count and albumin level.

Recent studies have reported that postoperative complications depend on liver function reserve and perioperative nutritional status [25–29]. Perioperative nutritional supplementation may thus reduce the risk of postoperative complications and shorten the duration of hospitalisation for patients with cancer requiring liver resection [30–32]. Hsieh et al. reported that postoperative nutritional support could contribute to the reduction of pulmonary complications, recovery of liver function, and shortened duration of hospitalisation among adult liver donors [33]. In addition, with high-risk hepatectomy for hepato-biliary cancer, synbiotics reportedly contribute to reductions in postoperative infectious complications [34]. Such beneficial effects would presumably involve correction of intestinal microbial imbalances induced by surgical stress. Furthermore, nutritional intervention could improve prognosis through improvements in the tolerance of patients for chemotherapy and surgical feasibility [30, 35].

In addition to the predictive value of postoperative complications, PNI was indicated as a potential prognostic indicator. Adjuvant chemotherapy could provide potential survival benefits in subgroups of patients exhibiting increased risk, such as patients with ICC showing advanced tumours or positive lymph node metastasis [36, 37]. Our results suggest that induction rates for adjuvant chemotherapy are reliant on PNI and postoperative complications. This may be one reason why low PNI and postoperative complications contribute to worsened oncological outcomes. However, in terms of the presence or absence of adjuvant chemotherapy, the prognosis of PNI< 50 was poor and could be considered an independent prognostic factor. Given the limited efficacy of surgery alone, multidisciplinary treatment with an appropriate combination of chemotherapy appears important for ICC. Various conditions result in the definition of ‘not optimally resectable’ cases with borderline disease; in such case, preoperative chemotherapy should be considered rather than up-front surgery with or without adjuvant chemotherapy. PNI could be useful in selecting patients for whom such preoperative chemotherapy, as well as nutritional therapy interventions, may hold promise.

### Limitations

As this study deliberately selected patients who had undergone curative surgical resection for ICC, we might have excluded patients with advanced ICC who did not undergo surgery because of tumour aggressiveness. The PNI in such patients would be expected to be low.

## Conclusions

Preoperative PNI can be used both as a predictive marker for the risk of severe postoperative complications and as a prognostic marker. PNI could reflect potential cancer progression that is difficult to determine using conventional diagnostic methods. Adequate nutritional intervention for patients with low PNI in the perioperative phase could contribute to further improvements in surgical outcomes and survival for patients with ICC.

## Supplementary Information

**Additional file 1: Supplementary Fig. 1.** a. Frequency of postoperative infectious complications in patients with hilar-type ICC (*n*=111). b. Frequency of postoperative infectious complications in patients who received bile duct resection (*n*=82).

**Additional file 2: Supplementary Fig. 2.** a. Kaplan-Meier curve for postoperative overall survival. b. Kaplan-Meier curve for overall recurrence-free survival. c. Kaplan-Meier curves for postoperative recurrence-free survival, stratified by grade of postoperative complications. d. Kaplan-Meier curves for postoperative recurrence-free survival, stratified by PNI.

## Data Availability

The data that support the findings of this study are available from the Okayama study group of Hepatobiliary and Pancreatic surgery (OS-HBP) but restrictions apply to the availability of these data, which were used under license for the current study, and so are not publicly available. Data are however available from the authors upon reasonable request and with permission of OS-HBP.
