# Supplementary figures and images for: Preoperative prognostic nutritional index predicts postoperative infectious complications and oncological outcomes after hepatectomy in intrahepatic cholangiocarcinoma
Source: BMC Cancer. 2021 Jun 16;21:708. doi: 10.1186/s12885-021-08424-0 (PMC8207701; doi:10.1186/s12885-021-08424-0)

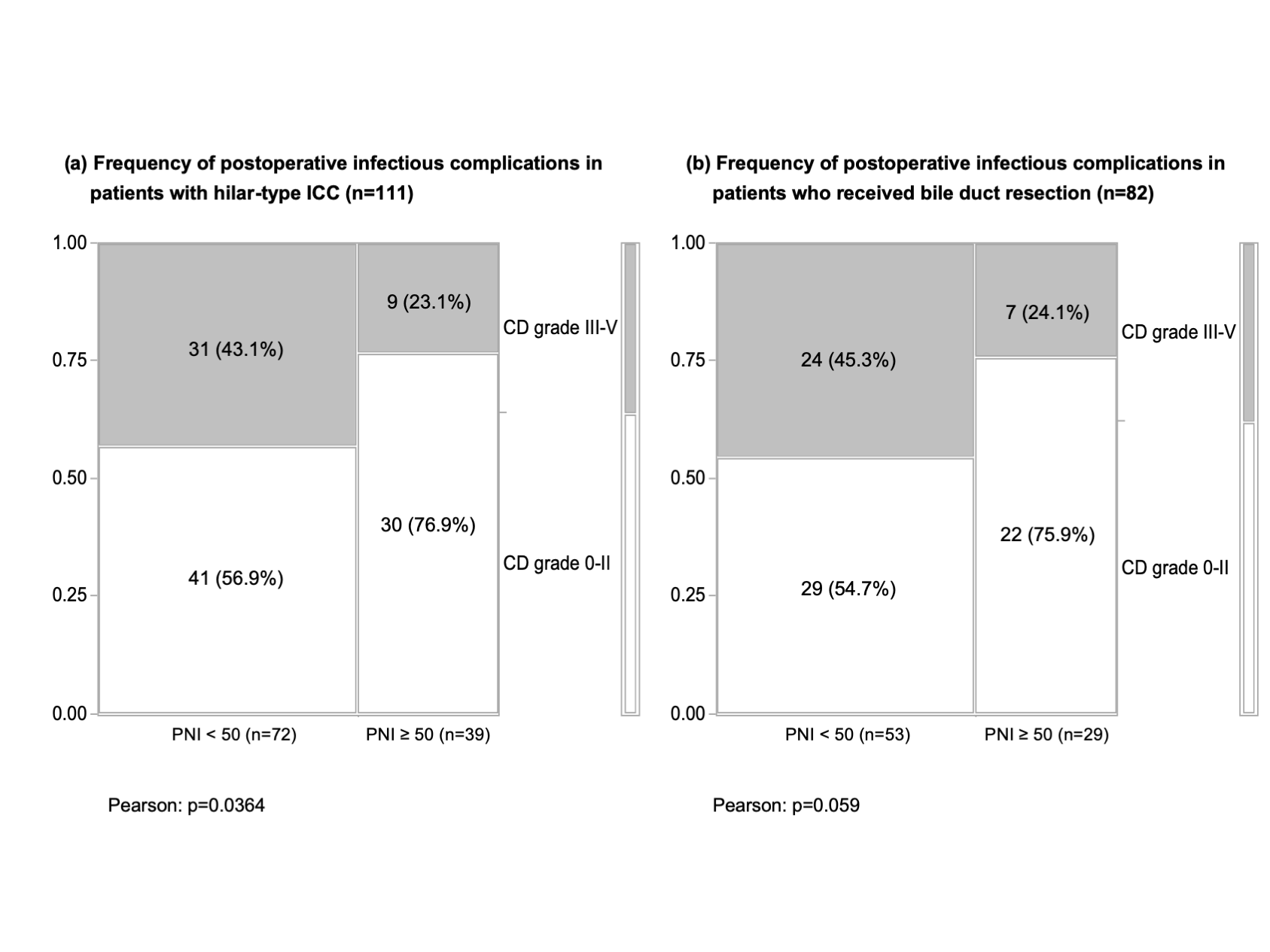

Supplement: Supplementary file 1 — Additional file 1: Supplementary Fig. 1. a. Frequency of postoperative infectious complications in patients with hilar-type ICC (n=111). b. Frequency of postoperative infectious complications in patients who received bile duct resection (n=82). [file 12885_2021_8424_MOESM1_ESM.tiff]

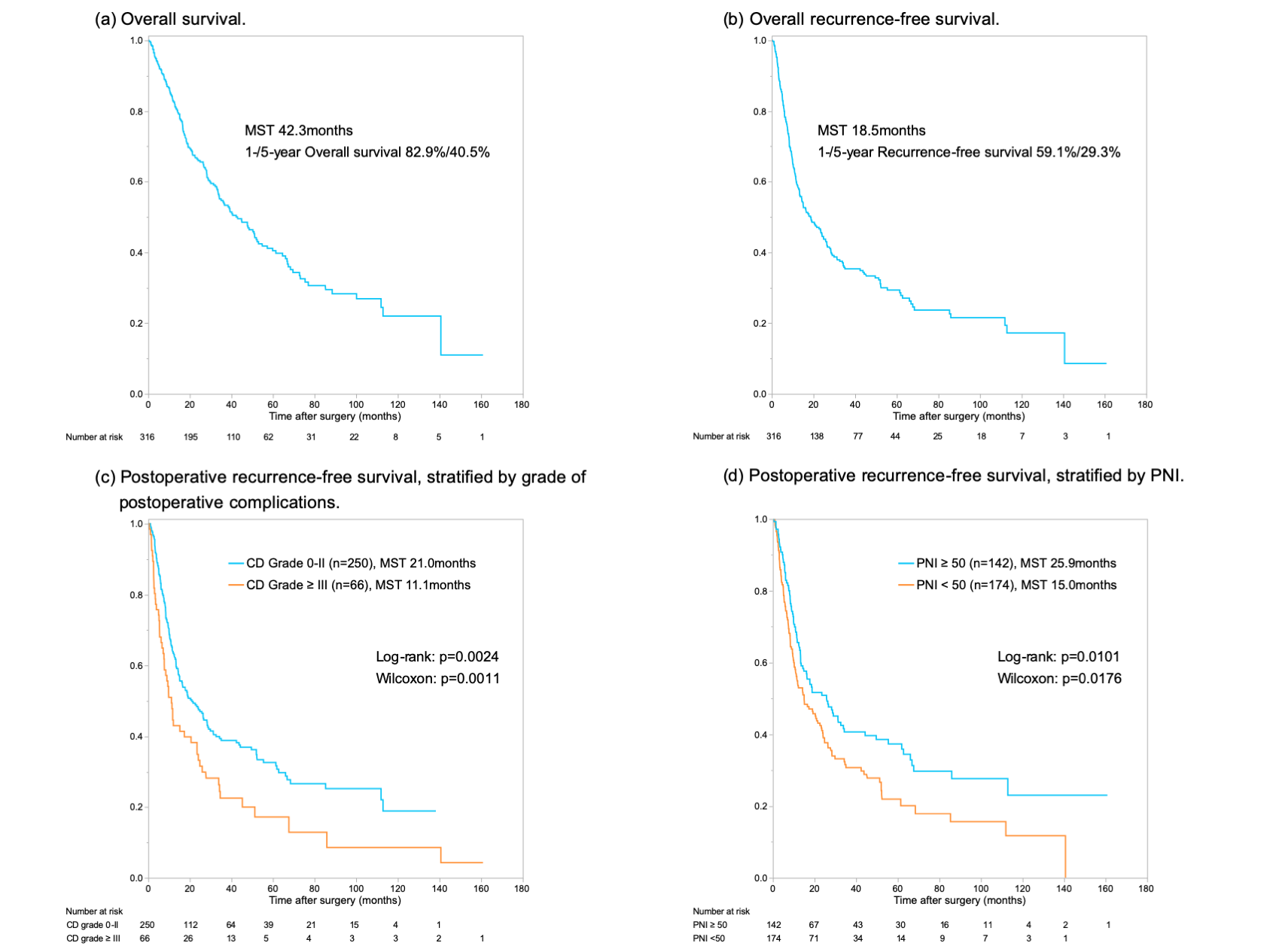

Supplement: Supplementary file 2 — Additional file 2: Supplementary Fig. 2. a. Kaplan-Meier curve for postoperative overall survival. b. Kaplan-Meier curve for overall recurrence-free survival. c. Kaplan-Meier curves for postoperative recurrence-free survival, stratified by grade of postoperative complications. d. Kaplan-Meier curves for postoperative recurrence-free survival, stratified by PNI. [file 12885_2021_8424_MOESM2_ESM.tiff]
